# Composite Adsorbent “1-Ethyl-3-methylimidazolium Acetate [EMIM] [Ac] into Mesoporous Silica Gel” for Adsorption Heat Storage

**DOI:** 10.3390/ma19051016

**Published:** 2026-03-06

**Authors:** Angelo Freni, Elisa Passaglia, Emilia Bramanti, Silvia Pizzanelli, Roberto Spiniello, Francesca Nardelli, Luigi Calabrese, Stefano De Antonellis, Giorgio Tomaino, Alejandro Jose Di Cicco

**Affiliations:** 1 Istituto di Chimica dei Composti OrganoMetallici, Consiglio Nazionale Delle Ricerche, Via G. Moruzzi 1, 56124 Pisa, Italy; elisa.passaglia@pi.iccom.cnr.it (E.P.); emilia.bramanti@pi.iccom.cnr.it (E.B.); silvia.pizzanelli@pi.iccom.cnr.it (S.P.); roberto.spiniello@pi.iccom.cnr.it (R.S.); luigi.calabrese@unime.it (L.C.); stefano.deantonellis@polimi.it (S.D.A.); 2 Department of Chemistry and Industrial Chemistry, University of Pisa, 56124 Pisa, Italy; francesca.nardelli@unipi.it; 3 Department of Engineering, University of Messina, 98166 Messina, Italy; 4 Department of Energy, Politecnico di Milano, 20156 Milan, Italy; giorgio.tomaino@polimi.it (G.T.); alejandrojose.dicicco@polimi.it (A.J.D.C.)

**Keywords:** adsorption, heat storage, ionic liquids, 1-ethyl-3-methylimidazolium acetate, composite adsorbent, mesoporous silica gel

## Abstract

The aim of this work is to prepare and characterize a composite adsorbent comprising the hydrophilic ionic liquid 1-ethyl-3-methylimidazolium acetate [EMIM-Ac] composite supported on mesoporous silica gel for application in adsorption heat storage systems. Water adsorption/desorption isotherms were measured gravimetrically at T = 40, 50, 70 °C across a relative humidity (RH) range of 0–0.8, demonstrating a high adsorption capacity (up to 0.71 g/g at 50 °C and RH = 0.8, for a 50 wt % [EMIM-Ac] loading). Full process reversibility and negligible ad/desorption hysteresis were also verified. Thermal stability of the prepared silica/[EMIM-Ac] composites was confirmed up to approximately T = 200 °C. Structural stability of samples subjected to repeated ad/desorption aging cycles was verified via FT-IR, High-Resolution Solid-State NMR, and Time-Domain NMR spectroscopy. Finally, the thermodynamic analysis based on adsorption experimental data indicated that the silica/[EMIM-Ac] composite is highly suitable for adsorption heat storage, providing a volumetric density of 600–920 MJ/m^3^ at regeneration temperatures below 100 °C.

## 1. Introduction

Within the wide class of thermochemical energy storage systems, adsorption heat storage is a promising technology due to its high energy density, environmental compatibility, flexibility and system efficiency [1]. This technology enables effective utilization of low-grade heat (temperatures generally between 70 and 150 °C), such as solar thermal energy or industrial waste heat [2]. The operating principle of an adsorption heat storage system is based on a reversible physical reaction which stores thermal energy using a working pair consisting of a solid sorbent (e.g., zeolites, silica gels) and a vapor (typically water). During the charging phase, the external heat source drives an endothermic desorption process, separating the adsorbate from the adsorbent. The discharging phase is initiated by the subsequent exothermic adsorption process that releases the stored heat. A key advantage of this technology is the almost negligible heat loss over extended periods, as the energy remains stored as chemical potential. Moreover, the working pairs used, such as water and silica gel, are typically inexpensive, non-toxic, and widely available. Current R&D efforts focus on increasing volumetric energy density, reducing system costs and optimizing the adsorbent material performance, which is the core element of the heat storage system [3].

Necessary characteristics for adsorption materials are high adsorption capacity in the typical heat storage operating conditions, low regeneration temperature, high ad/desorption rate, increased thermal conductivity, high stability and relatively low cost [4]. With water being the most common working fluid employed in many applications, different options have been examined, including silica gel, commercial and modified aluminosilicate zeolites (A, X, Y) [5], aluminophosphates, Metal–Organic Frameworks (MOFs) [6] and composite adsorbents consisting in a inorganic salt (e.g., CaCl_2_, LiCl, MgSO_4_) impregnated into a host matrix (mesoporous silica gel, carbons, clays) [7].

Recently, a new promising class of composite adsorbents consisting of ionic liquids (ILs) supported within a mesoporous host matrix (e.g., silica gels, silicalites) has emerged [8]. These novel composites exhibit interesting water adsorption properties for applications in adsorption cooling and desalination [9], as well as moisture removal systems [10,11]. The high affinity of specific ILs for water vapor allows for significantly higher uptake capacity and lower regeneration temperatures compared to traditional materials [12]. Confining the IL within a high-surface-area support material ensures IL containment and enhanced thermal stability during continuous cycling [13,14]. Furthermore, the high surface area allows improved mass transport kinetics and results in enhanced thermal properties of the composite, increasing overall performance and durability. The mechanism of water adsorption in these composites is generally a combination of physical adsorption on the host surface and absorption into the IL layer. This combination addresses the limitations of traditional adsorbents (e.g., lower capacity of silica gel or poor long-term stability of salt hydrates) [15].

Following this innovative approach, the primary objective of the present work is the preparation of the composite adsorbent: 1-Ethyl-3-methylimidazolium acetate ([EMIM-Ac]) impregnated into mesoporous silica gel. This specific room-T liquid IL has been specifically selected due to its stability and strong hydrophilic character [16,17,18], whereas mesoporous silica gel was selected due to its low regeneration temperature, affordability and commercial availability, good adsorption kinetics, and non-toxicity [19].

The scientific objectives of this work are to perform a comprehensive characterization of the silica/[EMIM-Ac] composite’s adsorption capacity, structural properties, and long-term durability, as well as to conduct a thermodynamic evaluation to determine its theoretical thermal energy storage efficiency. Specifically, a gravimetric apparatus was employed to measure water ad/desorption equilibrium and perform repeated ad/desorption aging cycles. Thermal stability of the composite was studied by TGA analysis, whereas the structure of unaged and aged silica/IL sample was investigated by Fourier Transform-Infrared (FT-IR), High-Resolution Solid State (SS-NMR) and Time-domain (TD-NMR) spectroscopies. Finally, the evaluation of thermal energy storage performance was carried out by a thermodynamic analysis based on experimental adsorption data.

## 2. Materials and Methods

### 2.1. Preparation of Silica/[EMIM-Ac] Composite Adsorbent

**Scheme 1:**
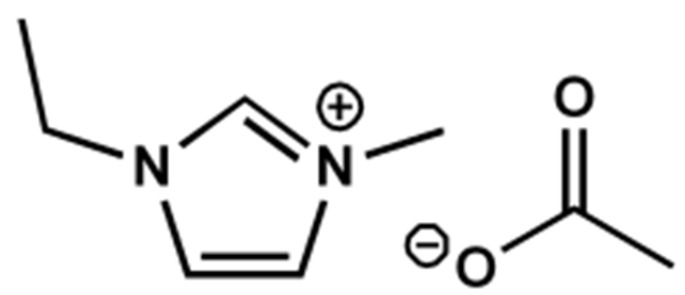
Chemical structure of the [EMIM] cation and [Ac] anion of the studied ionic liquid.

The ionic liquid [EMIM-Ac] (Scheme 1), Emim-Ac ionic liquid with a purity > 98% was purchased from Proionic GmbH (Grambach, Austria). Mesoporous Silica Gel was obtained from Sorption Technologies GmbH (Mönchengladbach, Germany).

[EMIM-Ac] and mesoporous silica gel main properties are reported in Table 1 and Table 2.

**Scheme 2:**
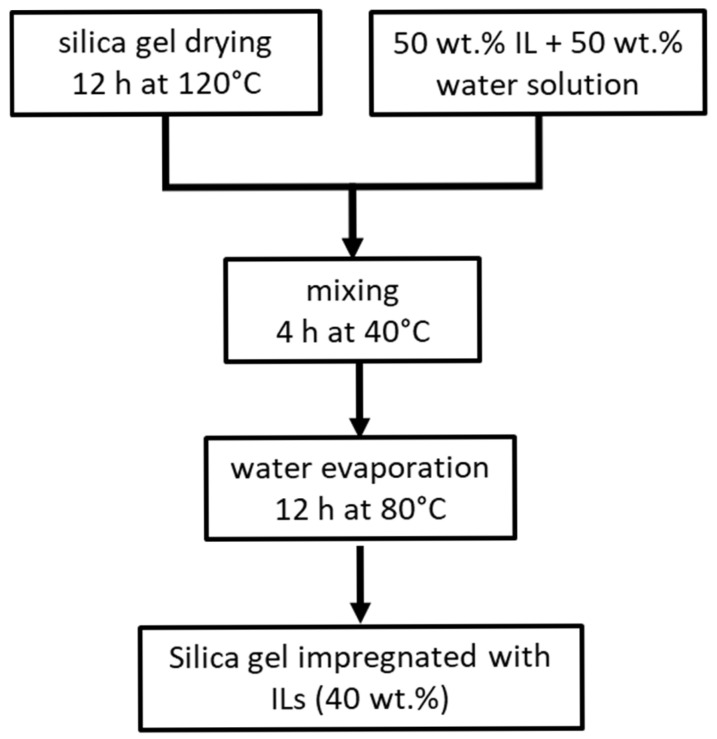
Silica/[EMIM-Ac] composite preparation procedure.

As reported in Scheme 2, the preparation of the silica/[EMIM-Ac] composite first required drying the mesoporous silica at 120 °C for 12 h. Subsequently, it was mixed with an aqueous solution of the ionic liquid in a 1:1 mass ratio (50 wt.% IL, 50 wt.% water). The mixture was maintained at 40 °C for 4 h to ensure homogeneous impregnation of the solution within the silica pores. Water was gently evaporated from the composite by heating at 80 °C for 12 h, resulting in a 40 wt.% concentration of the ionic liquid. Main thermophysical properties of the prepared composite adsorbent are reported in Table 3. For comparison, composite materials with ionic liquid mass fractions of 30% and 50% wt. % were prepared using a similar procedure.

### 2.2. Water Adsorption Isotherms by Gravimetric Adsorption Apparatus

Water adsorption isotherms were measured by means of a gravimetric adsorption apparatus (Aquadyne DVS analyzer, Boynton Beach, FL, USA). The instrument allows the measurement of adsorption isotherms by supplying a dry/wet carrier gas. Relative humidity (RH) is managed through a PID controller by mixing a stream of pure nitrogen with a stream of humidified nitrogen. The temperature is controlled by appropriate heating and cooling of the walls of the test chamber. The reference mass of the material *m_ref_* (anhydrous condition) was obtained in a nitrogen atmosphere at 80 °C (atmospheric pressure). Experimental uncertainties of measured quantities are the following: temperature ±0.2 °C; RH ±1% at 40 °C; weight ±1.0 μg plus 0.001% of suspended mass (measured sample and small glass container). Isotherm water vapor adsorption tests were conducted on small adsorbent composite samples (typically 50–200 mg) at 40 °C, 50 °C and 70 °C in the RH range 0–80%, representative of the typical operating range of adsorption heat storage systems. Considering the aforementioned information, the maximum uncertainty of the water uptake (*w* = *m*/*m_ref_* − 1) due to weight measurement is 0.01.

Aging tests were conducted using the same instrument to determine the stability of the prepared composite. Specifically, the sample was subjected to 100 adsorption/desorption cycles at a constant temperature (T = 40 °C) and varying RH. Each cycle had a duration of 60 min, involving a switch every 30 min between 5% RH and 80% RH.

### 2.3. TG Analysis

Thermogravimetric analysis was performed using a Seiko SII TG/DTA 7200 Exstar (Chiba, Japan) instrument. The measurement was conducted under an inert nitrogen atmosphere (200 mL/min) from 30 to 700 °C, utilizing an alumina pan and a heating rate of 10 K/min. Preliminary heating in an oven at 100 °C for 8 h was conducted to remove any moisture adsorbed in the samples.

### 2.4. FTIR Spectroscopy

Infrared spectra were recorded in reflectance mode by using a Perkin–Elmer Frontiers FTIR Spectrophotometer (Seer Green, Beaconsfield, UK), equipped with a universal attenuated total reflectance (ATR) accessory and a triglycine sulphate TGS detector. Three replicates for each sample were performed after background acquisition. For each sample, 32 scans were recorded, averaged and Fourier transformed to produce a spectrum with a nominal resolution of 4 cm^−1^.

### 2.5. SSNMR Spectroscopy

#### 2.5.1. High-Resolution SSNMR

Experiments were conducted on a Bruker AVANCE NEO 500 MHz NMR spectrometer (Rheinstetten, Germany) using a 4 mm probe operating at 99.35 MHz and 500.13 MHz on ^29^Si and ^1^H, respectively. The ^1^H 90° pulse was 2.9 μs. The ^1^H spectra were recorded using the single pulse sequence, using a recycle delay of 1 s.

The ^1^H-^29^Si CP-MAS NMR spectra were collected using the ramped CP sequence [23], where the ^1^H RF-field amplitude was linearly ramped from 50% to 100% of its nominal value. Spectra were acquired with a recycle delay of 1 s, a contact time of 5 ms, and by accumulating 12,000 transients. Both the ^1^H and ^29^Si spectra were recorded at a spinning rate of 5 kHz.

#### 2.5.2. TD-NMR

^1^H TD-NMR measurements were performed at a Larmor frequency of 20.8 MHz using a Niumag permanent magnet (Suzhou, China) interfaced with a Stelar PC-NMR console (Mede, Pavia, Italy). A 5 mm probe was used with a ^1^H 90° pulse duration of 3 μs for all samples. Carr–Purcell–Meiboom–Gill (CPMG) experiments were acquired to obtain ^1^H relaxation curves. 64 transients were accumulated using a recycle delay of 0.5 and 1 s and an echo delay of 50 and 200 μs for confined [EMIM-Ac] and pure [EMIM-Ac], respectively, and acquiring 2000 data points. CPMG relaxation curves were analyzed by a discrete approach using a non-linear least square fitting procedure implemented in the Mathematica^®^ environment.

## 3. Results and Discussion

### 3.1. Water Adsorption Isotherms

A preliminary water adsorption test was carried out on silica/[EMIM-Ac] composite samples with different ionic liquid mass fractions (30%, 40% and 50% wt.) to identify an optimal material composition.

**Figure 1:**
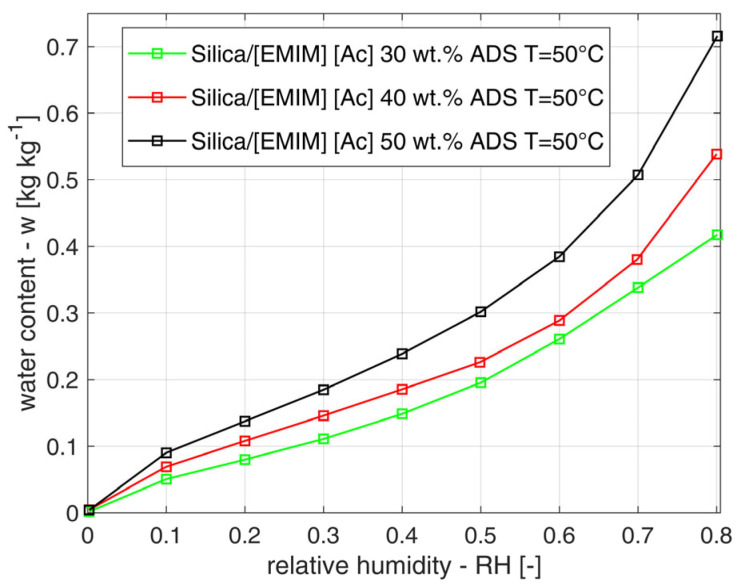
Water adsorption isotherm at 50 °C measured for composite “[EMIM-Ac] in mesoporous silica gel” with different weight fractions.

The results reported in Figure 1 show that an increase in IL content leads to higher water adsorption capacity, with the 50 wt.% sample exhibiting the best uptake performance. However, high concentrations of the hygroscopic phase may lead to leakage of the ionic liquid from the porous matrix, potentially affecting mass transfer kinetics and cyclic stability. For this reason, at this research step, the 40 wt.% composition was selected as the best compromise between adsorption capacity, pore accessibility and operational reliability and was therefore chosen for further in-depth analysis.

**Figure 2:**
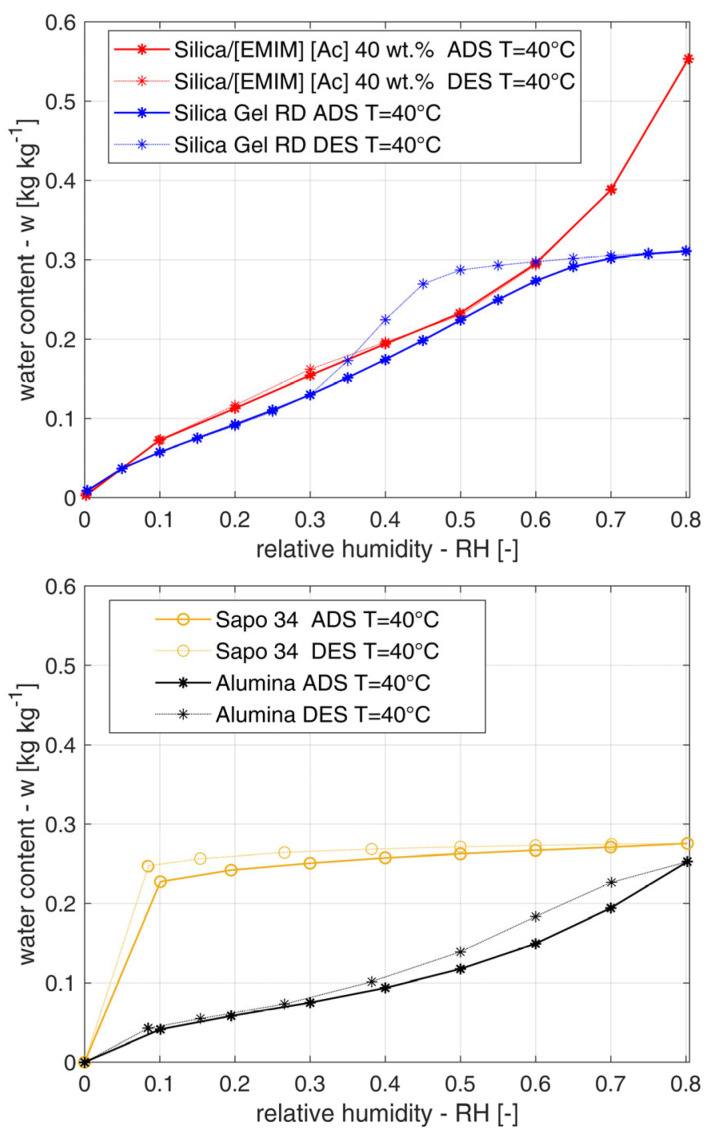
Water ad/desorption isotherm at 40 °C measured for: (**top**) the composite “[EMIM-Ac] 40 wt.% in mesoporous silica gel” and a standard microporous RD silica gel; (**bottom**) Sapo 34 powder and activated alumina beads.

Figure 2 shows the water ad/desorption isotherm (T = 40 °C) measured in the RH range 0–80% for the composite silica/[EMIM-Ac] 40 wt.% in comparison with standard microporous RD silica gel beads, activated alumina beads and Sapo 34 powder.

The results achieved showed that the silica/[EMIM-Ac] demonstrates high adsorption capacity specifically at elevated RH levels, reaching 0.54 g/g at 80% RH. This characteristic is highly attractive for diverse applications, including air treatment, desalination, and thermal energy storage. It is important to highlight that the composite demonstrated nearly negligible hysteresis and full ad/desorption process reversibility, indicating that the ionic liquid remains highly accessible within the silica pores and that the desorption process is not hindered by strong chemical bonding. The observed lack of hysteresis may not solely indicate intrinsic thermodynamic reversibility but could partially depend on the liquid nature of the ionic liquid absorbent. Compared to commercial microporous RD silica gel beads and activated alumina beads, the silica/[EMIM-Ac] shows higher sorption capacity, particularly at RH higher than 60%. Instead, by comparing the isotherm of Sapo-34 with that of silica/[EMIM-Ac], it emerges that the former material is more effective when regeneration occurs at RH < 10%, but less effective when regeneration and adsorption take place at RH > 10%. Therefore, in this case, the optimal material selection depends on the operating conditions.

**Figure 3:**
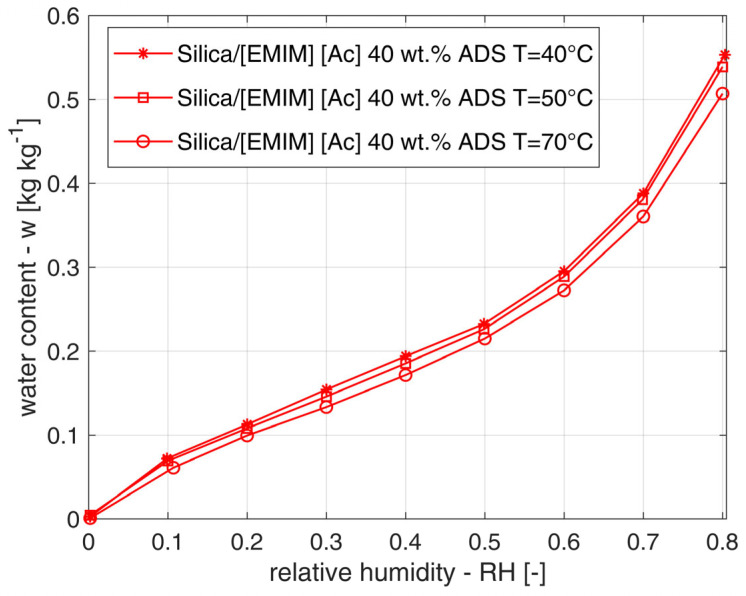
Water adsorption isotherms at 40 °C, 50 °C and 70 °C measured for the composite “[EMIN-AC] 40 wt.% in mesoporous silica gel”.

Figure 3 shows the water adsorption isotherms of the silica/[EMIM-Ac] 40 wt.% composite measured at 40 °C, 50 °C and 70 °C. As expected, increasing temperature leads to a systematic decrease in the equilibrium water uptake at a given RH, while the overall shape of the isotherms remains essentially unchanged.

### 3.2. Aging Test by Repeated Ad/Desorption Cycles

**Figure 4:**
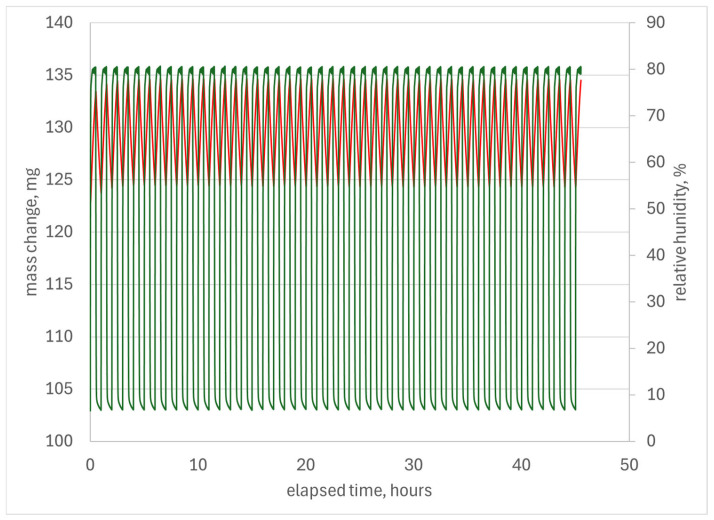
Aging test and RH profile of the composite silica/[EMIM-Ac] 40 wt.%.

Figure 4 shows the water content and RH profiles measured for the silica/[EMIM-Ac] 40 wt.% composite over 20 representative adsorption/desorption cycles. The analysis of these profiles demonstrates that the sample mass variation remained constant throughout the entire test duration. Furthermore, the water adsorption capacity of the composite material was found to be unaltered after the completion of 100 total cycles. In fact, once the conditions have stabilized, the amount of water absorbed/desorbed in each cycle is 10.03 mg ± 0.03 mg, without any decreasing trend.

This finding indicates that the composite material has not undergone any structural deterioration or performance degradation during the aging protocol, thereby confirming the long-term stability and robustness of the composite under continuous cyclical humidity stress.

### 3.3. TG Analysis

**Figure 5:**
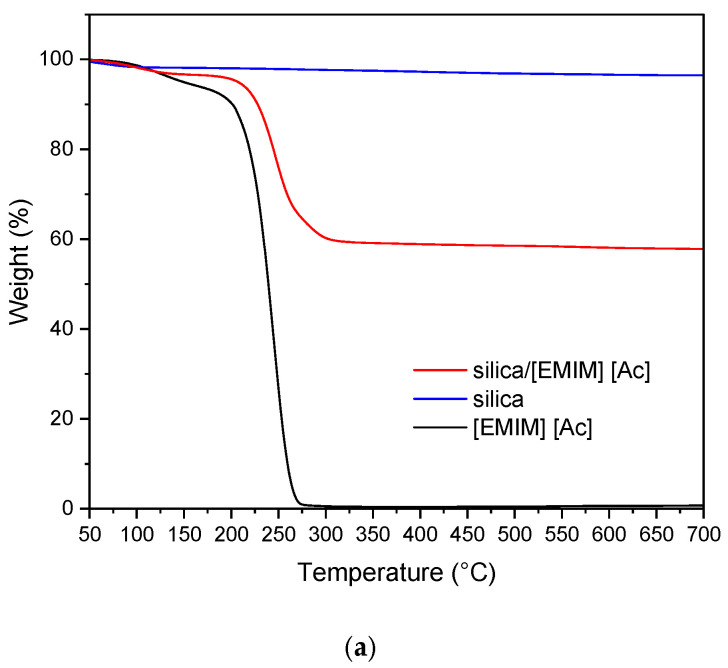

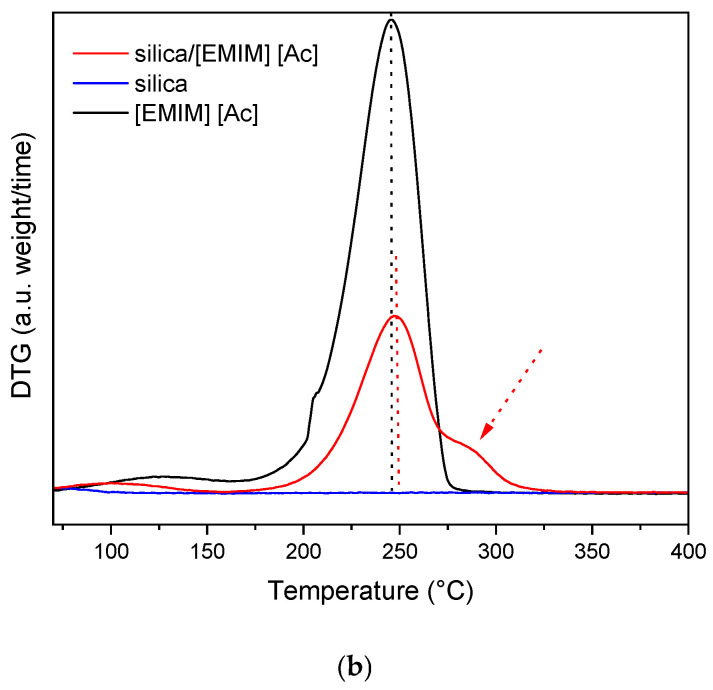
TG (**a**) and DTG (**b**) curves of the composite silica/[EMIM-Ac] 40 wt.%, as well as of the pure mesoporous silica gel and IL [EMIM-Ac].

Figure 5 shows TG (Figure 5a) and DTG (Figure 5b) curves of the composite silica/[EMIM-Ac] 40 wt.%, as well as of the pure mesoporous silica gel and pure ionic liquid compound.

The pure [EMIM-Ac]IL presumably contains a residual amount of moisture (or other volatile species) of approximately 5 wt.% despite the thermal conditioning. The onset of thermal degradation, determined as the intersection between the baseline weight and the tangent to the steepest slope of the mass-loss curve, occurs at 212 °C, in good agreement with literature data [18]. After impregnation onto silica, the silica/[EMIM-Ac] composite exhibits an increased degradation onset temperature (T_onset_ = 226 °C), corresponding to a shift of about 14 °C. This shift indicates that confinement of the IL within the porous silica matrix enhances its thermal stability (Figure 5a).

This stabilizing effect is further supported by DTG analysis (Figure 5b): the silica/[EMIM-Ac] sample shows a slight shift of T_infl_, defined as the temperature at the maximum degradation rate (DTG peak). Moreover, an additional DTG peak at approximately 290 °C is clearly observed (red arrow in Figure 5b). This feature suggests that immobilization of the IL on silica alters the degradation pathway, leading to the formation of a fraction that decomposes at higher temperatures, suggesting/proving the interaction between IL and the porous inorganic substrate.

Overall, TGA analysis demonstrates that the silica/[EMIM-Ac] composite exhibits thermal stability up to approximately 200 °C under the investigated conditions, which is fully compatible with the intended applications in adsorption-based heat transformation and storage. Finally, the residual mass at 700 °C confirms an IL loading of about 40 wt.%.

### 3.4. ATR-FTIR Analysis

**Figure 6:**
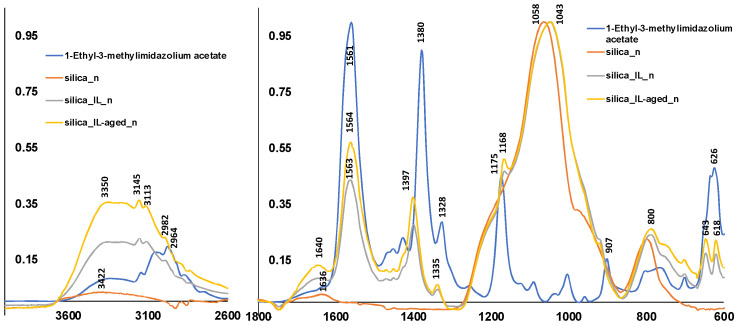
Normalized FT-IR spectra of 1-Ethyl-3-methylimidazolium acetate (IL, blue line), silica gel (orange line), silica gel/IL composite (grey line) and aged silica gel/IL composite (yellow line), (silica gel 60–/IL 40 wt.%).

Figure 6 shows the ATR-FTIR spectra of fresh and aged silica/[EMIM-Ac] 40 wt.% composite. The spectrum of the pristine mesoporous silica gel host matrix and of the pure [EMIM-Ac] are also reported for comparison.

In the FTIR spectrum of silica gel, the broad bands in the range 3600–2600 cm^−1^ and at 1636 cm^−1^ are assigned to the stretching and bending vibrations of silanol groups (Si–OH), respectively. The bands found at 1058 and 800 cm^−1^ are the characteristic anti-symmetric and symmetric stretching modes Si–O–Si) of [SiO_4_] units, respectively. A shoulder observed at 974 cm^−1^ is due to the Si–OH stretching mode [24,25].

In FTIR spectrum of -Ethyl-3-methylimidazolium acetate (IL), we observe the -CH_2_, CH_3_ stretching modes in the 3200–2800 cm^−1^ region, the -CH_2_, CH_3_ bending modes at 1328 cm^−1^, the vibrational mode of the C-N bending of the ring at 1561 cm^−1^ of the anion, and the symmetric and asymmetric stretching of COO^−^ at 1380 and 1590 cm^−1^, respectively, of the cation, the latter convoluted with the absorption of C-N stretching mode of the ring. The OH stretching vibrations of water are also observed in the 3600–2600 cm^−1^ region.

The spectroscopic data indicate a chemical–physical sorption of IL onto silica gel. The chemical interaction is demonstrated by the shift in several bands of silica gel and IL. The symmetric stretching of COO^−^ at 1380 shifts to 1396 cm^−1^, as well as the -CH_2_, CH_3_ bending modes at 1328 cm^−1^ shift to 1168 cm^−1^. The peak at 626 cm^−1^ is not described in the literature; it could be related to the structure of IL. In the silica gel/IL system, this peak is split into two peaks at 643 and 618 cm^−1^.

The vibrational analysis did not show any significant variation in the material after aging, except for about 30% decrease in the OH band area, likely due to the removal of bond water.

### 3.5. NMR Spectroscopy

#### 3.5.1. Characterization of Confined IL

**Figure 7:**
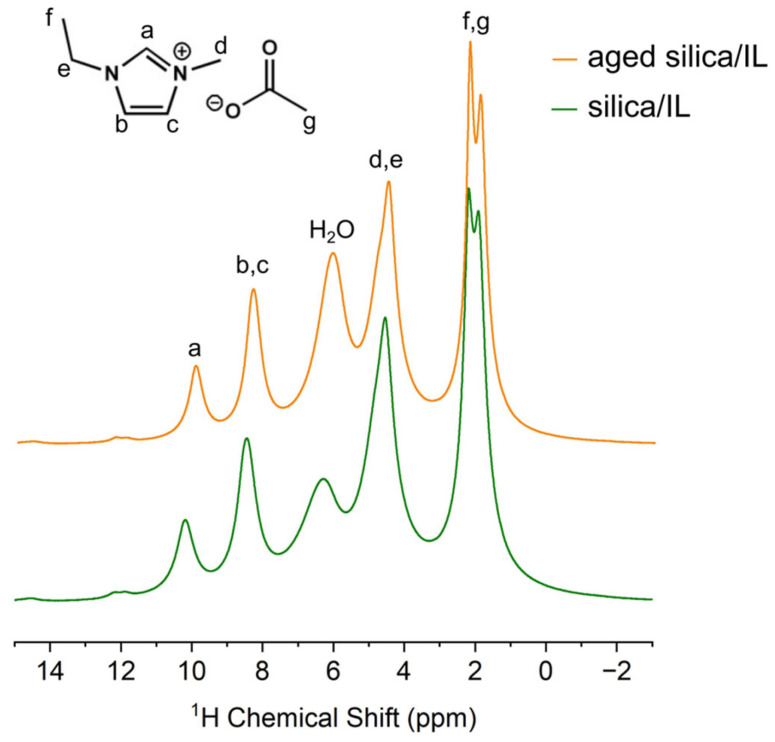
^1^H MAS NMR spectra of the unaged and aged silica/IL samples with signal assignment.

^1^H MAS NMR was used to assess whether the IL undergoes degradation upon aging. Figure 7 shows the ^1^H MAS NMR spectra of the fresh and aged silica/IL samples. In the fresh sample, the signals at 1.9, 2.2, 4.5, 8.5, and 10.2 ppm are assigned to the different IL protons, as reported in Figure 7 and according to literature data on the pure IL [26], whereas the signal at approximately 6 ppm is attributed to water. The IL signals are preserved in the aged sample and maintain the relative intensities observed in the fresh one, although the chemical shifts in the imidazolium protons are slightly shifted. This effect might be ascribed to a slight change in the H-bond network, possibly induced by a higher water content. Indeed, the ratio between the integral of the water signal and that of one of the signals due to the IL almost doubles when going from the fresh to the aged sample. Apart from this, no evidence of ionic liquid degradation is detected in the spectral analysis.

**Figure 8:**
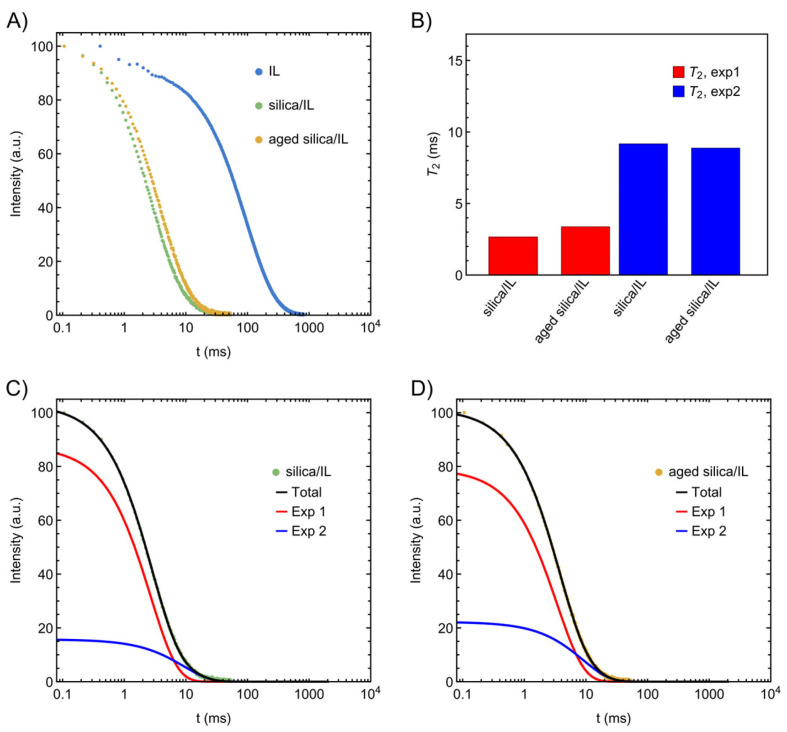
(**A**) ^1^H CPMG relaxation curves of the indicated samples. (**B**) Histogram of the *T*_2_ values obtained from the ^1^H FID analysis of the silica/IL and aged silica/IL composites. Analysis of ^1^H CPMG relaxation curves of unaged silica/IL composite (**C**) and aged silica/IL composite (**D**).

To characterize differences in molecular mobility of confined IL upon thermal aging, TD-NMR spectroscopy was employed to measure the transverse relaxation time, *T*_2_ of ^1^H nuclei. These relaxation times reflect the modulation of nuclear interactions, mainly ^1^H-^1^H dipolar couplings, due to molecular motion. Using a low-field NMR spectrometer, ^1^H CPMG relaxation curves were acquired (Figure 8A). The curves were reproduced using two exponential components, each defined by a weight and a *T*_2_ value (Figure 8C,D). The first component with a shorter *T*_2_ (Exp1) was ascribed to the IL, whereas the second component with a longer *T*_2_ value (Exp2), ascribable to more mobile molecules, was assigned to water. Comparable *T*_2_ values were found for both aged and unaged composites (Figure 8B), suggesting that thermal aging has a negligible impact on the dynamics of the IL confined in the silica matrix. However, the *T*_2_ values are significantly shorter than those of the bulk IL, confirming reduced mobility due to confinement. Notably, in the aged sample, the exponential component characterized by the longer *T*_2_ value exhibits a higher weight than in the fresh sample (22% and 15%, respectively), confirming the presence of a larger amount of water in the aged sample, as already observed by ^1^H MAS NMR.

Overall, these results indicate that the IL does not undergo appreciable degradation upon aging and that a consistent amount of water is absorbed by the ionic liquid because of aging.

#### 3.5.2. Characterization of the Silica Matrix

**Figure 9:**
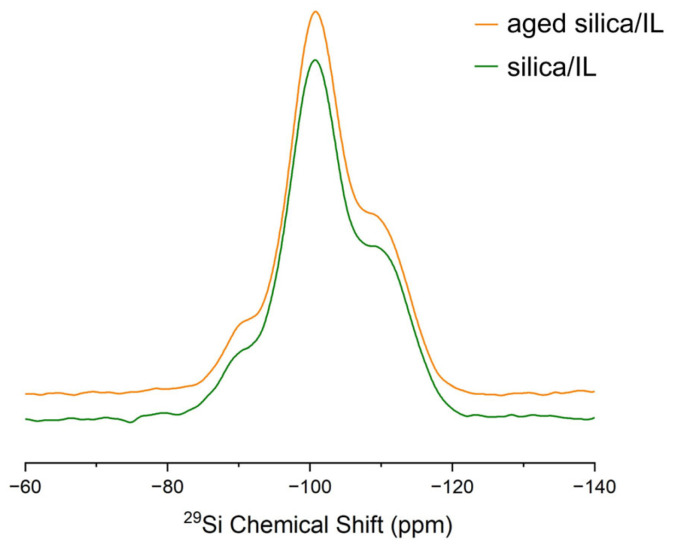
^1^H-^29^Si CP-MAS NMR spectra of the fresh and aged silica/IL samples.

^29^Si NMR was used to evaluate whether aging affects the speciation of silicon atoms in the matrix. The ^1^H-^29^Si CP-MAS NMR spectra of the fresh and aged silica/IL samples, shown in Figure 9, allowed silicon atoms in different chemical environments to be distinguished, namely Q^2^ (Si(OH)_2_(OSi)), Q^3^ (Si(OH)(OSi)_3_), and Q^4^ (Si(OSi)_4_) sites, which resonate at about −90, −100, and −110 ppm, respectively.

No significant differences were observed between the ^1^H-^29^Si CP-MAS NMR spectra of the fresh and aged silica/IL samples, indicating that aging does not affect the speciation of silicon atoms in the silica matrix.

## 4. Evaluation of Thermal Energy Storage Density

### 4.1. Adsorption Data Correlation and Interpolation

**Figure 10:**
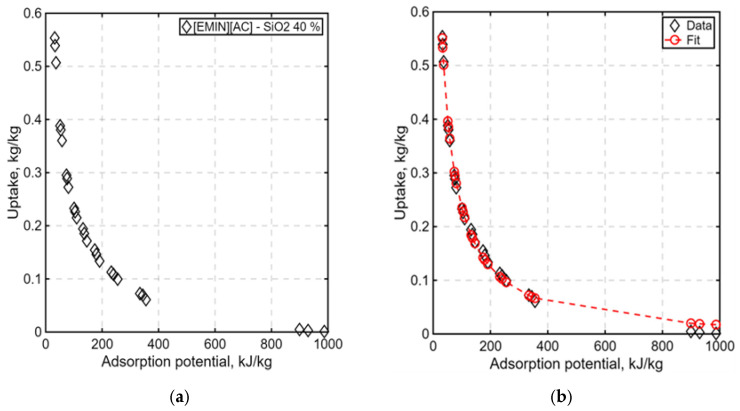
(**a**) Equilibrium adsorption uptake *w* as a function of the Dubinin–Polanyi potential *A* for the composite silica/[EMIM-Ac] 40 wt.%; (**b**) fitting of the experimental data by D-A approach.

**Figure 11:**
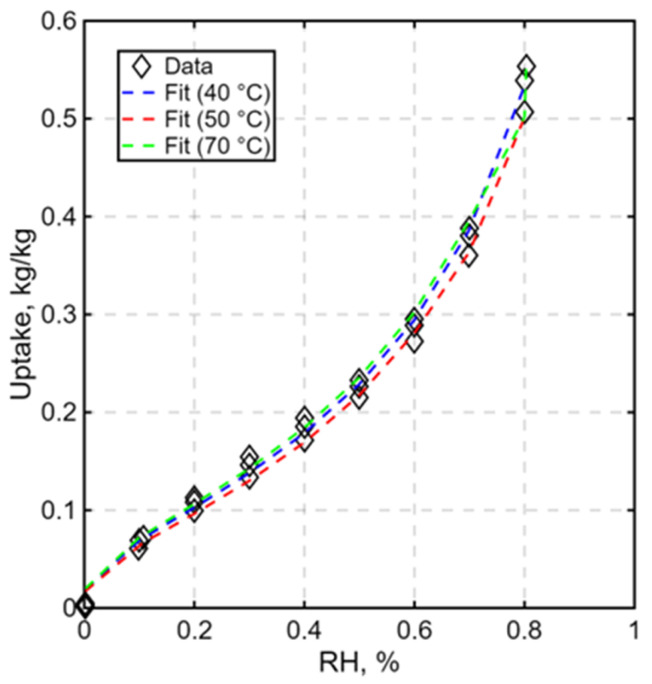
Comparison of experimental and calculated adsorption data using the D-A model for the composite “silica/[EMIM-Ac]/water” working pair.

A proper experimental adsorption data interpolation model was employed to perform a thermodynamic analysis and estimate the energy density increase achievable with the silica/IL composites compared to state-of-the-art values. Specifically, the description of the water adsorption curves under equilibrium conditions on the composite silica/[EMIM-Ac] 40 wt.% was obtained using the well-known Dubinin–Astakhov (D-A) thermodynamic correlation [27], based on Polanyi’s empirical potential theory. This theory considers the adsorption process to be analogous to the phenomenon of pore filling by condensation; therefore, we assume that the characteristic adsorption curve is independent of temperature. This specific assumption implies that the D-A model can be rigorously applied only to non-polar adsorbent solids (e.g., activated carbons). However, extensive literature demonstrates that this approach can also be applied with good accuracy to describe adsorption phenomena in systems with polar adsorbents (e.g., silica gel/water) [28]. The D-A model has also been successfully employed for unconventional adsorbents, including MOFs, SAPO zeolites, and composite materials impregnated with hygroscopic substances [28,29,30]. Therefore, it is possible to consider the D-A model as a useful engineering tool for the design and simulation of thermochemical energy storage processes. In particular, the D-A model allows for the description of adsorption isotherms under equilibrium conditions through the following correlation [31]:

(1) $$w=w_{0}\exp\left[-{\left(\frac{A}{E}\right)}^{n}\right]$$

where *w* (kg(kg) represents the uptake of adsorbed water vapor per unit mass of adsorbent material, *w*_0_ (kg(kg) represents the maximum adsorption capacity, *E* (kJ/kg) is a term that represents the characteristic energy of adsorption for a given adsorbate, and *n* is an empirical parameter that depends on the heterogeneity of the porous structure. Typically, the three terms *w*_0_, *E*, *n* are determined by fitting the experimental adsorption curves. The term A (kJ/kg) represents the Dubinin–Polanyi adsorption potential, defined as follows:

(2) $$A=RT\ln\frac{p_{s}(T)}{p}$$

where *R* (J/mol K) is the universal gas constant, *T* (K) is the adsorption temperature, *p_s_* (kPa) is the saturated vapor pressure at the adsorption temperature *T*, and *p* (kPa) is the actual water vapor pressure applied on the adsorbent material. The main advantage of the D-A approach is the possibility of describing the adsorption capacity *w* with a function represented by a single parameter (the adsorption potential, *A*), rather than two parameters (*p* and *T*), which offers clear computational advantages. The following Figure 10a shows the experimental temperature-independent characteristic curve defined as uptake *w* as a function of the Dubinin–Polanyi potential *A *(Equation (2)) for the composite silica/[EMIM-Ac] 40 wt.%, while Figure 10b presents the non-linear curve fit of the experimental data following the D-A approach, in order to determine the values of the parameters *w*_0_, *E*, *n*. The best-fit values are presented in the subsequent Table 4:

The following Figure 11 shows the comparison between the experimental adsorption curve and the curve calculated through fitting with the D-A model (Equation (1)), clearly highlighting excellent fitting in the temperature range 40–70 °C.

### 4.2. Thermal Energy Storage Performance Evaluation

**Figure 12:**
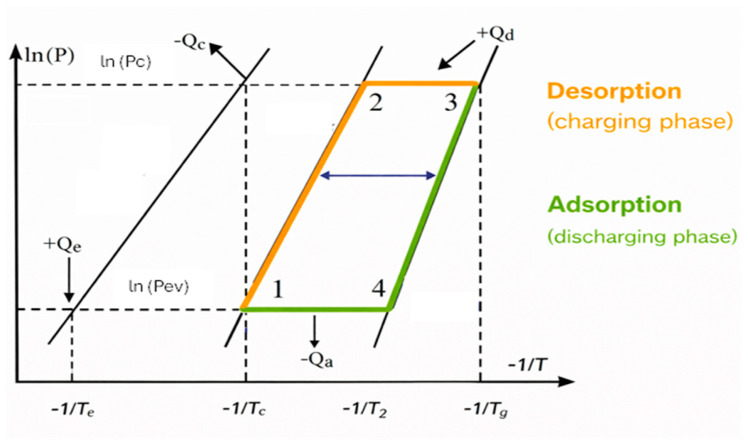
Heat storage adsorption cycle plotted on the Clapeyron diagram (*ln(p), T*).

Figure 12 shows a typical heat storage adsorption cycle plotted on an isosteric Clapeyron diagram (*ln(p)*, *T*). The working cycle can be divided into two phases:

(i) A charging phase, in which heat is supplied to the system (desorption), consisting of an isosteric heating (Sections 1 and 2 in the figure) and isobaric desorption (2–3) at the condenser pressure *p_c_*;

(ii) A discharging phase (adsorption) which consists of an isosteric cooling (Sections 3 and 4) and isobaric adsorption (4-1) at the evaporator pressure *p_ev_*.

The boundary conditions of the thermodynamic cycle are established by defining three thermal levels: *T_g_* = 90 °C—regeneration (desorption) temperature of the adsorbent during the discharge phase; *T_ads_* = 40 °C—temperature during the discharge phase; *T_ev_* = 15 °C—evaporation temperature.

**Figure 13:**
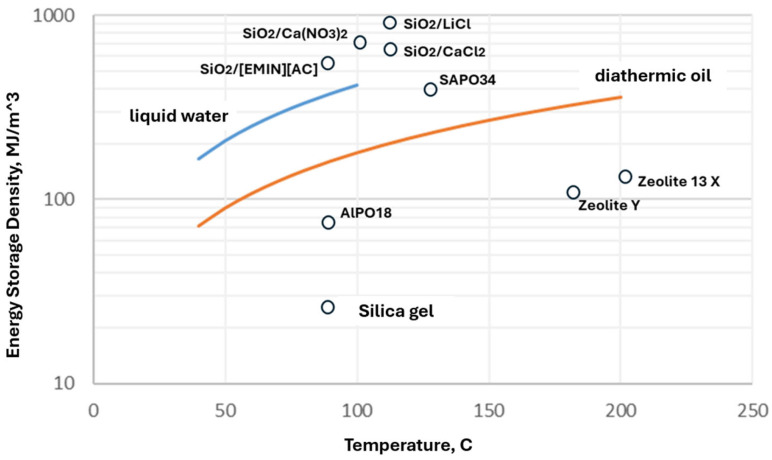
Thermal energy storage density determined for various adsorbent materials under the following operating conditions: *T_ev_* = 15 °C, *T_con_* = 35 °C, *T_ads_* = 40 °C, *T_de_*_s_ = 90–200 °C).

Considering the thermodynamic transformations presented in Figure 12, it is possible to define the energy storage density as:

(3) $$E=\frac{Q_{a}}{m_{ads}}$$

where *m_ads_* [kg] is the mass of the adsorbent and *Q_a_* [kJ/kg] is the heat extracted during discharging (adsorption process), the sum of the heat of adsorption plus the sensible heat contribution:

(4) $$Q_{a}=\int_{T_{i}}^{T_{e}}\left(\int_{w_{min}}^{w_{max}}{cp}_{ads}dw\right)dT+\int_{w_{min}}^{w_{max}}{∆H}_{a}dw$$

where *dw* is the cyclic water uptake, i.e., the difference between the maximum and minimum sorbate uptake during the cycle. *ΔH_a_* is the isosteric heat of adsorption (kJ/kg sorbate) required to desorb the adsorbate from the adsorbent, which can be estimated as follows [32]:

(5) $${∆H}_{a}=L+A$$

where *L* [kJ/kg] is the latent heat of vaporization for the water and *A* is the adsorption potential defined in Equation (2). In Figure 13, an evaluation of the volumetric energy density is performed under typical operating conditions for low/mid temperature thermal storage (*T_ev_* = 15 °C, *T_con_* = 35 °C, *T_ads_* = 40 °C, *T_des_* = 90–200 °C), based on literature data or experimentally measured adsorption data for various adsorbent materials.

The results generally highlight that the use of composite materials—consisting of hydrophilic ionic liquids or inorganic salts impregnated into porous solid matrices (mesoporous silica gel)—enables a volumetric thermal storage density in the range of 600–920 MJ/m^3^. This is significantly higher than the typical storage density of conventional adsorbent materials (zeolite 13X, Y, AlPO/SAPO34, silica gel) and higher than the sensible thermal storage density of commercial diathermic oils (300 MJ/m^3^) under comparable conditions. It is also highlighted that the composite developed in this work, consisting of [EMIM-Ac] impregnated in mesoporous silica gel, provides the greatest benefit in terms of energy density increase compared to state-of-the-art values for desorption temperatures below 100 °C. An additional benefit is the absence of corrosion or deliquescence issues that are typical of composite materials employing inorganic salts as hygroscopic substances.

## 5. Conclusions

In this study, a novel composite adsorbent was prepared by supporting the ionic liquid 1-ethyl-3-methylimidazolium acetate [EMIM-Ac] onto mesoporous silica gel. The resulting silica/[EMIM-Ac] composite demonstrated a superior water sorption capacity (up to 0.71 g/g at 80% RH for a 50 wt% IL loading) compared to other state-of-the-art adsorbents such as microporous RD silica gel, alumina and SAPO34 zeolite. Adsorption/desorption isotherms exhibited negligible hysteresis and full reversibility, which are important features for practical operation in adsorption cycles. TGA confirmed the composite thermal stability up to 200 °C, ensuring suitability for low-grade heat applications. Furthermore, FT-IR and SS-NMR analyses of aged samples verified that the IL maintains its structural integrity and remains stable within the mesoporous framework. Finally, a thermodynamic analysis based on experimental adsorption data revealed that the silica/[EMIM-Ac] composite can provide energy storage density of 600–920 MJ/m^3^ with a regeneration temperature below 100 °C, which is very attractive for designing compact and efficient Thermal Energy Storage (TES) systems driven by low-temperature heat sources.

## Figures and Tables

**Table 1:** Main properties of [EMIM-Ac] [20,21].

Property	Value
Density	1.1 g/cm^3^
Viscosity	91 mPa s
Molecular weight	170.21 g/mol
T decomposition	221 °C
T melting	<0 °C
T combustion	Not flammable
Vapor pressure	Negligible

**Table 2:** Main properties of mesoporous silica gel [22].

Property	Value
Grain size	0.125–0.4 mm
Specific surface area	267 m^2^/g
Specific pore volume	1.1 cm^3^/g
Average pore diameter	15.9 nm

**Table 3:** Main thermophysical properties of the composite silica/[EMIM-Ac] 40 wt.%.

	Density	Specific Heat	Thermal Conductivity
	g/cm^3^	kJ/(kg K)	W/(m K)
Mesoporous silica gel	0.70	0.85	0.17
[EMIM-Ac]	1.10	1.85	0.22
Silica/[EMIM-Ac] 40 wt.%	0.85	0.92	0.20

**Table 4:** Best-fit parameters of the Dubinin–Astakhov equation determined for the composite “silica/[EMIM-Ac] 40 wt.%/water” working pair.

	A	w_o_	E	*n*
	kJ/kg	kg/kg	kJ/kg	
Silica/[EMIM-Ac] 40 wt.%	30–990	7.1824	0.7092	0.2482

## Data Availability

The original contributions presented in this study are included in the article. Further inquiries can be directed to the corresponding author.

## Nomenclature

E	Characteristic energy of adsorption for a given adsorbate, kJ/kg
D-A	Dubinin–Astakhov model
A	Dubinin–Polanyi adsorption potential, kJ/kg
*n*	Empirical parameter
*E*	Energy storage density
FT-IR	Fourier Transform–Infrared spectroscopies
*Q* *a*	Heat exchanged during discharging/adsorption phase, kJ/kg
IL	Ionic Liquid
∆*Ha*	Isosteric heat of adsorption, kJ/kg
*L*	Latent heat of vaporization for the water, kJ/kg
m	Mass, kg
*w* *o*	Maximum uptake of adsorbed water vapor, kg/kg
P	Pressure
RH	Relative humidity, %
*c* *p*	Specific heat, kJ/kg/K
T	Temperature, °C
TGA	Thermogravimetric Analysis
TD-NMR	Time-domain Nuclear Magnetic Resonance spectroscopies
R	Universal gas constant, J/mol/K
*w*	Uptake of adsorbed water vapor, kg/kg
Subscripts:	
*a* *d* *s*	Adsorption
*a* *d* *s*	Adsorbent
*c*	Condenser
*c* *o* *n*	Condenser
*d* *e* *s*	Desorption
*e* *v*	Evaporator
*ref*	Reference
*g*	Regeneration
*s*	Saturated
